# Continuous manufacturing of lentiviral vectors using a stable producer cell line in a fixed-bed bioreactor

**DOI:** 10.1016/j.omtm.2024.101209

**Published:** 2024-02-09

**Authors:** Dale J. Stibbs, Pedro Silva Couto, Yasuhiro Takeuchi, Qasim A. Rafiq, Nigel B. Jackson, Andrea C.M.E. Rayat

**Affiliations:** 1 Department of Biochemical Engineering, University College London, Bernard Katz Building, Gower Street, London WC1E 6BT, UK; 2 Division of Infection and Immunity, University College London, Cruciform Building, Gower Street, London WC1E 6BT, UK; 3 Biotherapeutics and Advanced Therapies, Scientific Research and Innovation, Medicines and Healthcare Products Regulatory Agency, South Mimms, Potters Bar EN6 3QC, UK; 4 Cytiva, 5 Harbourgate Business Park, Southampton Road, Portsmouth PO6 4BQ, UK

**Keywords:** lentiviral vector, stable producer cell line, continuous bioprocessing, fixed-bed bioreactor, perfusion culture

## Abstract

Continuous manufacturing of lentiviral vectors (LVs) using stable producer cell lines could extend production periods, improve batch-to-batch reproducibility, and eliminate costly plasmid DNA and transfection reagents. A continuous process was established by expanding cells constitutively expressing third-generation LVs in the iCELLis Nano fixed-bed bioreactor. Fixed-bed bioreactors provide scalable expansion of adherent cells and enable a straightforward transition from traditional surface-based culture vessels. At 0.5 vessel volume per day (VVD), the short half-life of LVs resulted in a low total infectious titer at 1.36 × 10^4^ TU cm^−2^. Higher perfusion rates increased titers, peaking at 7.87 × 10^4^ TU cm^−2^ at 1.5 VVD. The supernatant at 0.5 VVD had a physical-to-infectious particle ratio of 659, whereas this was 166 ± 15 at 1, 1.5, and 2 VVD. Reducing the pH from 7.20 to 6.85 at 1.5 VVD improved the total infectious yield to 9.10 × 10^4^ TU cm^−2^. Three independent runs at 1.5 VVD and a culture pH of 6.85 showed low batch-to-batch variability, with a coefficient of variation of 6.4% and 10.0% for total infectious and physical LV yield, respectively. This study demonstrated the manufacture of high-quality LV supernatant using a stable producer cell line that does not require induction.

## Graphical abstract

**Figure:**
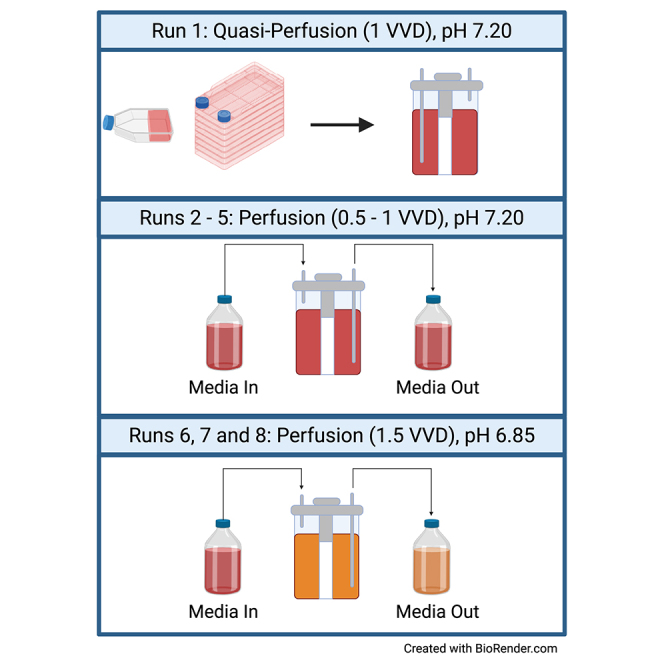

## Teaser

Rayat and colleagues developed a continuous process for producing high-quality LVs in a fixed-bed bioreactor using a stable producer cell line. The process was optimized by modulating the perfusion rate and lowering the culture pH. This provided extended vector production, improved reproducibility, and cost savings compared with transfection-based manufacturing.

## Introduction

Lentiviral vectors (LVs) efficiently can transduce both dividing and non-dividing cells and mediate transgene integration into the target cell genome, providing stable transgene expression.^1^ Their versatility is further enhanced by the ability to pseudotype them with various heterologous envelope glycoproteins, enabling the transduction of a wide range of cells.^2^

LVs are commonly used to genetically modify T cells to express chimeric antigen receptors (CARs) on their surface.^3^ These cells are then infused into the patient, killing cancerous cells by recognizing tumor-associated antigens.^4^ Hematopoietic stem cells (HSCs) are also frequently genetically modified using LVs to treat genetic diseases, such as sickle cell disease, adenosine deaminase-deficient severe combined immunodeficiency, metachromatic leukodystrophy, and Wiskott-Aldrich syndrome.^5^^,^^6^^,^^7^^,^^8^

The multiplicity of infection (MOI) is the LV-to-cell ratio used during transduction. Selecting the MOI requires consideration of transduction efficiency, gene expression level, safety concerns, cell viability, and cell heterogeneity. For CAR T cell manufacturing, the MOI is typically in the range of 1–10.^9^^,^^10^^,^^11^^,^^12^ In contrast, CD34^+^ HSCs are more resistant to LV-based gene modification, so high MOIs (∼100) or successive rounds of transduction are required to achieve the relevant product specifications.^8^^,^^13^^,^^14^^,^^15^

The dominant approach for manufacturing LVs is the chemical transfection of cells with plasmid DNA encoding the vector components.^16^ Transient transfection can achieve high titers once optimized and are flexible, allowing production to pivot to manufacture vectors with different transgenes and envelope proteins. However, challenges include the need to optimize the transfection conditions, difficulty in scaling, and the high cost of the plasmid DNA and transfection reagents.^17^^,^^18^

Stable producer cell lines that express all the required genes for LV production represent a more scalable and cost-effective manufacturing approach to transient transfection.^17^^,^^19^ However, constructing these cell lines is challenging as HIV-1 protease and vesicular stomatitis virus glycoprotein G (VSV-G) envelope protein exhibit cytotoxic and cytostatic effects on the producer cells. Inducible stable cell lines have been developed to provide controlled expression of cytotoxic proteins by adding an inducer or removing a suppressor.^19^ Alternatively, continuous packaging cell lines that sustain the expression of all vector components without cytotoxic limitations can be used.^20^^,^^21^^,^^22^^,^^23^^,^^24^ This work uses the WinPac-RDpro-GFP cell line that constitutively expresses third-generation RD114-Pro pseudotyped LVs with a GFP marker, which has been used to produce vectors in static vessels.^24^^,^^25^^,^^26^

LV production for pre-clinical and early-stage clinical studies usually occurs in culture flasks. Increasing production capacity generally involves augmenting the adherent culture surface with supplementary vessels.^27^ However, manual maintenance of flasks is labor intensive, time consuming, and prone to contamination.^16^ Multilayer systems facilitate scaling production by offering interconnected, vertically stacked growth surfaces that eliminate the need for additional incubator space.^28^^,^^29^ Static systems face various limitations, such as the inability to monitor and control essential process parameters, sub-optimal oxygenation, limited scalability, and susceptibility to contamination. Therefore, transitioning to bioreactors is essential to achieve scalable LV manufacturing.

Fixed-bed bioreactors provide an alternative to traditional surface-providing culture systems.^30^ An immobilized, three-dimensional matrix of porous microfiber carriers facilitates cell adhesion and expansion. They have a smaller footprint than flasks and can be scaled with iCELLis (Cytiva) and Scale-X (Univercells) platforms, providing surface areas of 0.53–500 m^2^ and 2.4–600 m^2^, respectively.^31^^,^^32^^,^^33^^,^^34^^,^^35^ Considering the predominant use of adherent producer cell lines, there has been significant interest in employing fixed-bed bioreactors for LV production.^16^ Continuous bioprocessing provides several benefits, including cost reduction, increased productivity, reduced facility footprint, multiproduct production capabilities, adaptability to changing demands, and improved product quality.^36^^,^^37^^,^^38^

This work evaluated the impact of modulating perfusion rate on LV titers, supernatant quality, and culture conditions. Subsequently, the effect of reducing the culture pH from 7.20 to 6.85 was assessed as mildly acidic culture conditions have previously been associated with increased LV titers.^31^^,^^33^^,^^39^ Transfection-based vector production provides inherent variability, and transitioning to stable cell line-based production could improve process robustness.^40^ Given this, the batch-to-batch variability of the established process was evaluated by performing three independent runs.

## Results

### Cell growth kinetics and distribution

The LV manufacturing process in the iCELLis Nano bioreactor lasted 10 days. WinPac-RDpro-GFP cells were inoculated on day 0 at a seeding density of 3 × 10^4^ cells cm^−2^. Medium exchange for nutrient supply, removal of inhibitory metabolites, and LV harvest began 2 days after seeding. As detailed in Table 1, the medium exchange was performed using quasi-perfusion at 1 vessel volume per day (VVD) (run 1) or perfusion at a fixed rate between 0.5 and 2 VVD (runs 2–8). Quasi-perfusion is an approach to simulate perfusion operation by undertaking medium exchange by batch, rather than continuously, at defined intervals. In this case, medium exchange was performed once a day at the scale equivalent to 1 vessel volume. The culture pH was maintained at 7.20 ± 0.05, except in runs 6, 7, and 8, which were reduced to 6.85 ± 0.05 from day 2.

**Table 1:** Description of iCELLis Nano bioreactor runs for continuous LV production process development

Run number	Medium exchange strategy	Culture pH Setpoint	Goal
1	Quasi-Perfusion – 1 VVD	7.20 ± 0.05	Transfer process from static culture
2	Continuous – 0.5 VVD	7.20 ± 0.05	Perfusion rate investigation
3	Continuous – 1 VVD	7.20 ± 0.05	Perfusion rate investigation
4	Continuous – 1.5 VVD	7.20 ± 0.05	Perfusion rate investigation
5	Continuous – 2 VVD	7.20 ± 0.05	Perfusion rate investigation
6	Continuous – 1.5 VVD	6.85 ± 0.05	Reduced culture pH
7	Continuous – 1.5 VVD	6.85 ± 0.05	Repeat of run 6
8	Continuous – 1.5 VVD	6.85 ± 0.05	Repeat of run 6

The bioreactor was seeded with 3 × 10^4^ cells cm^−2^ on day 0, with medium exchange commencing 2 days after seeding for 8 days.

The iCELLis Nano bioreactor fixed-bed comprises three-dimensional polyethylene terephthalate (PET) macro-carriers. After inoculating, the cells attach or become entrapped in the macro-carriers. Figure S1 shows that 80% cell attachment was achieved across the eight runs in 4–6 h. Once 80% attachment was achieved, the linear speed within the bioreactor was reduced from 2 cm s^−1^ to 1 cm s^−1^.

**Figure 1:**
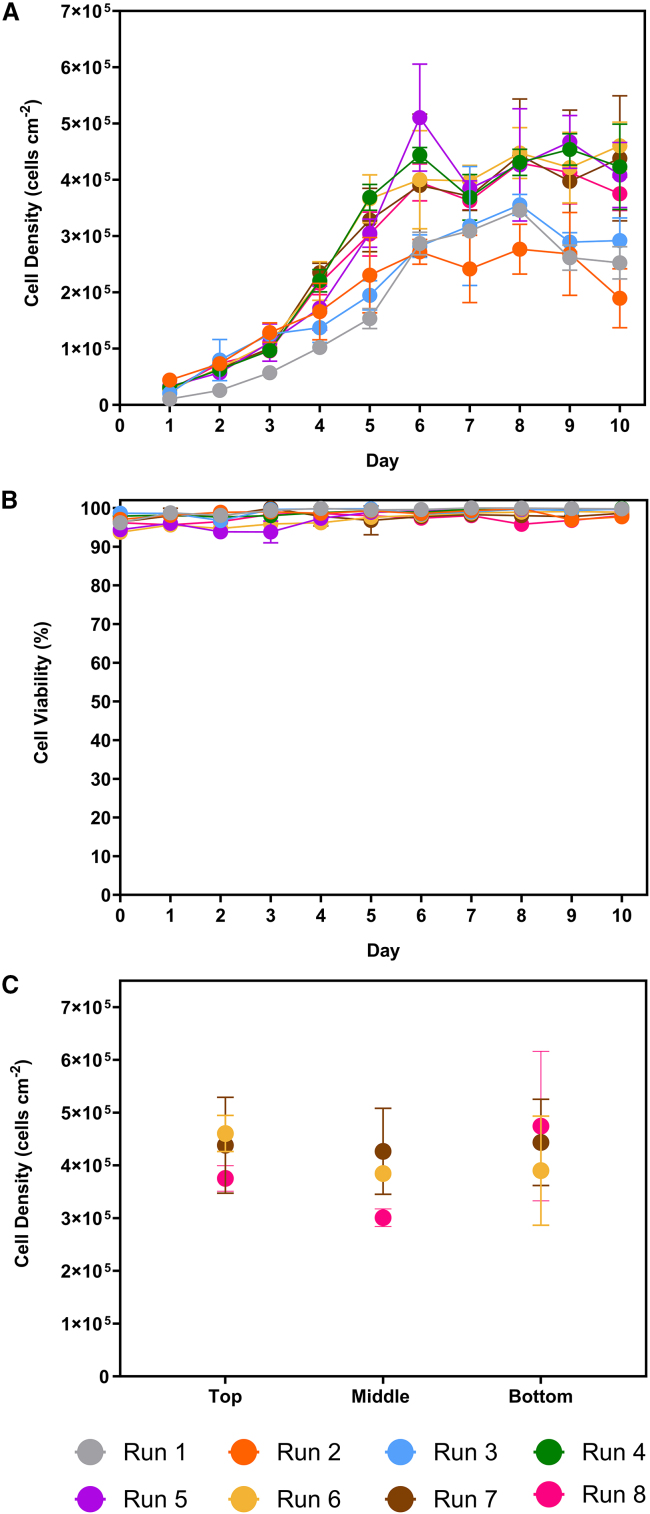
Growth kinetics and cell distribution WinPac-RDpro-GFP (A) cell density and (B) cell viability in the iCELLis Nano bioreactor runs. Three macro-carriers were samples from each bioreactor. (C) Distribution of WinPac-RDpro-GFP cells in the iCELLis Nano bioreactor fixed-bed at day 10 of runs 6–8 as measured from the top, middle, and bottom macro-carriers. Points represent mean value ± 1 SD (n = 3).

The bioreactor design allowed the removal of macro-carriers from the top of the fixed bed, allowing offline monitoring of cell density throughout the process. Figure 1A shows this was observed daily by sampling the top carriers and counting the lysed nuclei. The cell growth in the bioreactor exhibited a sigmoid pattern. The maximum cell densities were the lowest during runs 1, 2 and 3, where they varied between (2.77 ± 0.36) × 10^5^ and (3.55 ± 0.15) × 10^5^ cells cm^−2^. The remaining processes achieved between (4.30 ± 0.09) × 10^5^ and (5.10 ± 0.15) × 10^5^ cells cm^−2^. Figure 1B shows that cell viability was maintained above 90% in all runs.

The trends in Figure 1A were reflected in the specific growth rates, fold increases, and doubling times presented in Table 2. The highest doubling times of 44.2, 45.3, and 45.4 h occurred in runs 1, 2 and 3, respectively. Doubling times were lower in runs 4–8, ranging between 35.2 and 38.9 h. The calculated population doublings were lowest in runs 1 and 2 at 3.3 and 3.2, respectively. All the remaining processes exhibited higher doublings, ranging between 3.7 and 4.1.

**Table 2:** Specific growth rate, doubling time and population doublings of WinPac-RDpro-GFP cells during the iCELLis Nano bioreactor runs

Run number	Specific growth rate (h^−1^)	Doubling time (h)	Population doublings
1	0.016	44.2	3.3
2	0.015	45.3	3.2
3	0.015	45.4	3.7
4	0.019	37.0	3.9
5	0.020	35.2	4.1
6	0.018	38.5	3.7
7	0.018	38.9	3.7
8	0.018	38.7	3.7

The bioreactor was seeded with 3 × 10^4^ cells cm^−2^ on day 0, with medium exchange commencing 2 days after seeding for 8 days. Three macro-carriers were sampled from each bioreactor.

The fixed-bed (2 cm, low compaction) with a surface area of 0.53 m^2^ was disassembled after repeat runs 6–8. Figure 1C shows the cell distribution at the top, middle, and bottom of the fixed bed during these runs. The cell distribution at the bottom was (3.7 ± 8.2)% lower than at the top of the bed across these runs.

### Metabolite concentrations

**Figure 2:**
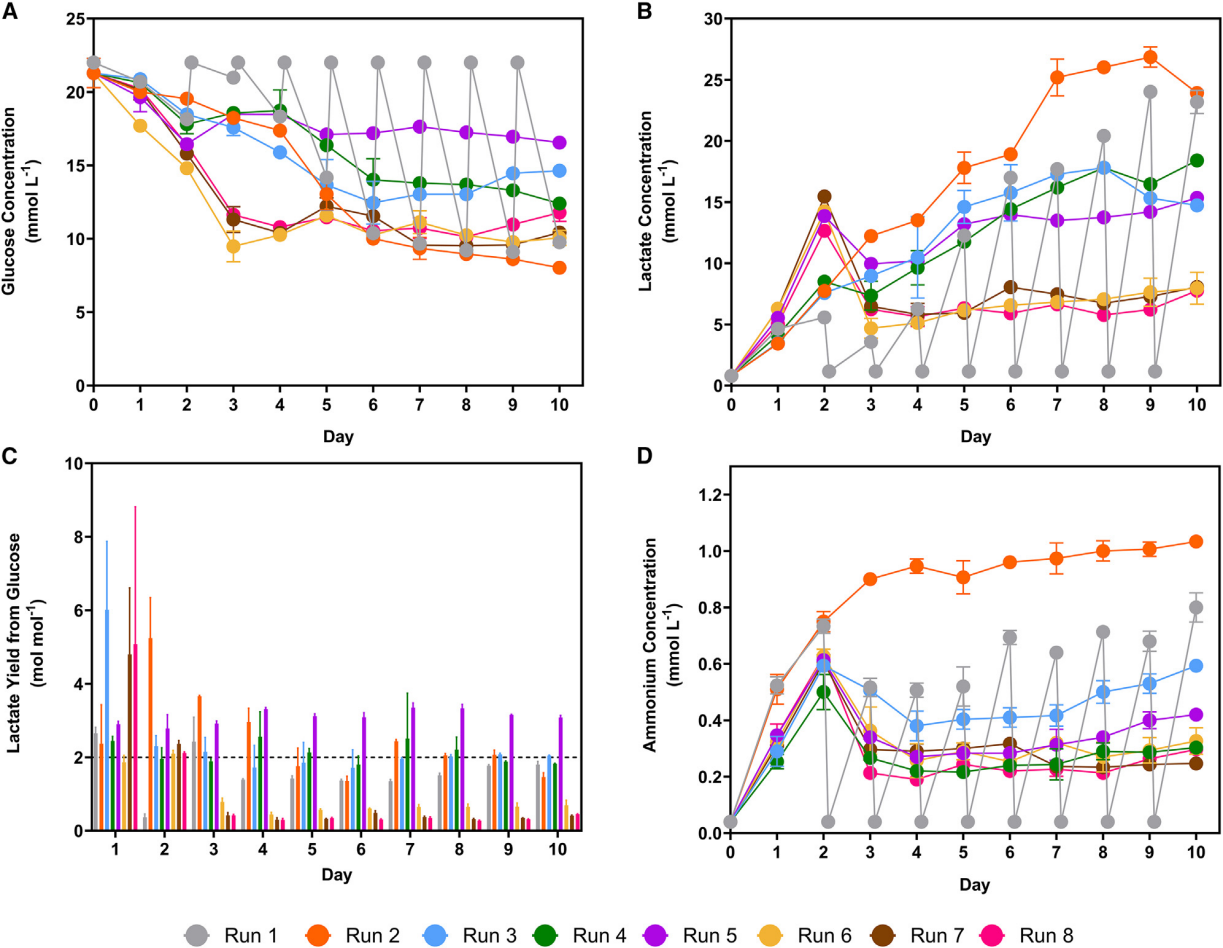
Metabolite concentrations Daily offline measurement of (A) glucose, (B) lactate, and (D) ammonium during the iCELLis Nano bioreactor runs. Points represent mean value ± 1 SD (n = 1). (C) The lactate yield from glucose for each iCELLis Nano bioreactor runs from days one to ten. Bars represent mean value ± 1 SD (n = 3). The 2 mol mol^−1^ reference line indicates the maximum theoretical yield of lactate from glucose from anaerobic glycolysis.

As depicted in Figure 2A, the glucose concentration was monitored throughout the bioreactor runs. In the initial 2 days, all runs exhibited a decline in concentration from (22.02 ± 0.12) mmol L^−1^ in fresh medium to (17.18 ± 1.49) mmol L^−1^. Runs 6–8, where the culture pH was decrease to 6.85 on day 2, experienced a sharp decrease in glucose concentration until day 3, reaching (10.81 ± 1.15) mmol L^−1^. Subsequently, the glucose concentration stabilized, plateauing at (10.62 ± 0.82) mmol L^−1^. Conversely, runs operated at a pH of 7.20 exhibited a gradual decrease in glucose concentration and demonstrated a general trend of higher concentrations being maintained with increased perfusion rates.

Lactate accumulation was observed during the bioreactor runs, as shown in Figure 2B. Before medium exchange commenced on day two, concentrations increased to (10.70 ± 3.52) mmol L^−1^. The lowest perfusion rate (0.5 VVD) exhibited the highest lactate concentrations, surpassing 20 mmol L^−1^ from day 6 onward. Accumulation above 20 mmol L^−1^ was observed from day 8 in the quasi-perfusion process at 1 VVD. The 1, 1.5, and 2 VVD perfusion processes displayed similar concentration profiles. Notably, runs conducted at a pH of 6.85 demonstrated lower lactate concentrations. Between days 2 and 10, lactate concentrations in these runs averaged (6.60 ± 1.00) mmol L^−1^.

The lactate yield from glucose was calculated to assess the metabolic efficiency of the WinPac-RDpro-GFP cell line, as depicted in Figure 2C. In the initial two days, the calculated yield occasionally exceeded the maximum theoretical value of 2 mol mol^−1^. During the exponential growth phase, the yields were around 2 mol mol^−1^, except in runs 6–8, where these decreased to (0.45 ± 0.15) mol mol^−1^.

As shown in Figure 2D, ammonium concentrations showed a notable increase in all runs during the initial two days of culture before initiating medium exchange. Concentrations rose from (0.04 ± 0.01) mmol L^−1^ to an average of (0.63 ± 0.08) mmol L^−1^ across all 8 runs. The highest ammonium concentrations were observed in runs 1 and 2, reaching (0.80 ± 0.04) mmol L^−1^ and (1.03 ± 0.01) mmol L^−1^, respectively, on day 10. Perfusion culture effectively reduced ammonium accumulation, with an average concentration of (0.36 ± 0.11) mmol L^−1^ on day 10 of runs 3–8.

### LV titers

Table 3 presents the physical and infectious LV titers and the physical-to-infectious particle ratios. The initial quasi-perfusion run at 1 VVD yielded infectious and physical LV yields of 3.75 × 10^4^ transducing units per cm^2^ (TU cm^−2^) and 8.74 × 10^6^ physical vector particles per cm^2^ (vp cm^−2^), respectively. The physical-to-infectious particle ratio was 226.

**Table 3:** Infectious and physical LV yields obtained from the iCELLis Nano bioreactor runs

Run number	Total TU	Total vp	Total TU cm^−2^	Total vp cm^−2^	Ratio vp:TU
1	1.99 × 10^8^	4.49 × 10^10^	3.75 × 10^4^	8.47 × 10^6^	226
2	7.20 × 10^7^	4.75 × 10^10^	1.36 × 10^4^	9.86 × 10^6^	659
3	3.28 × 10^8^	4.94 × 10^10^	6.19 × 10^4^	9.32 × 10^6^	151
4	4.17 × 10^8^	7.77 × 10^10^	7.87 × 10^4^	1.47 × 10^7^	186
5	3.98 × 10^8^	6.42 × 10^10^	7.52 × 10^4^	1.21 × 10^7^	161
6	4.82 × 10^8^	6.52 × 10^10^	9.10 × 10^4^	1.23 × 10^7^	135
7	5.24 × 10^8^	6.35 × 10^10^	9.89 × 10^4^	1.20 × 10^7^	121
8	5.65 × 10^8^	7.89 × 10^10^	1.07 × 10^5^	1.49 × 10^7^	140

For each bioreactor run, infectious and physical titers were determined with six and three technical repeats, respectively.

After establishing the process in quasi-perfusion mode, the impact of modulating the perfusion rate between 0.5 and 2 VVD was investigated. The lowest infectious titer of 1.36 × 10^4^ TU cm^−2^ was observed at 0.5 VVD, while higher rates of 1 and 1.5 VVD increased infectious titers to 6.19 × 10^4^ TU cm^−2^ and 7.87 × 10^4^ TU cm^−2^, respectively. No further increase in infectious LV was observed at 2 VVD compared to with the 1.5 VVD process. The lowest physical LV yields were observed at 0.5 and 1 VVD at 9.86 × 10^6^ vp cm^−2^ and 9.32 × 10^6^ vp cm^−2^, respectively. Higher perfusion rates increased yields of 1.47 × 10^7^ vp cm^−2^ and 1.21 × 10^7^ vp cm^−2^ at 1.5 and 2 VVD, respectively. The highest physical-to-infectious particle ratio of 659 was observed at 0.5 VVD, while the 1, 1.5, and 2 VVD ratios were 151, 186, and 161, respectively.

**Figure 3:**
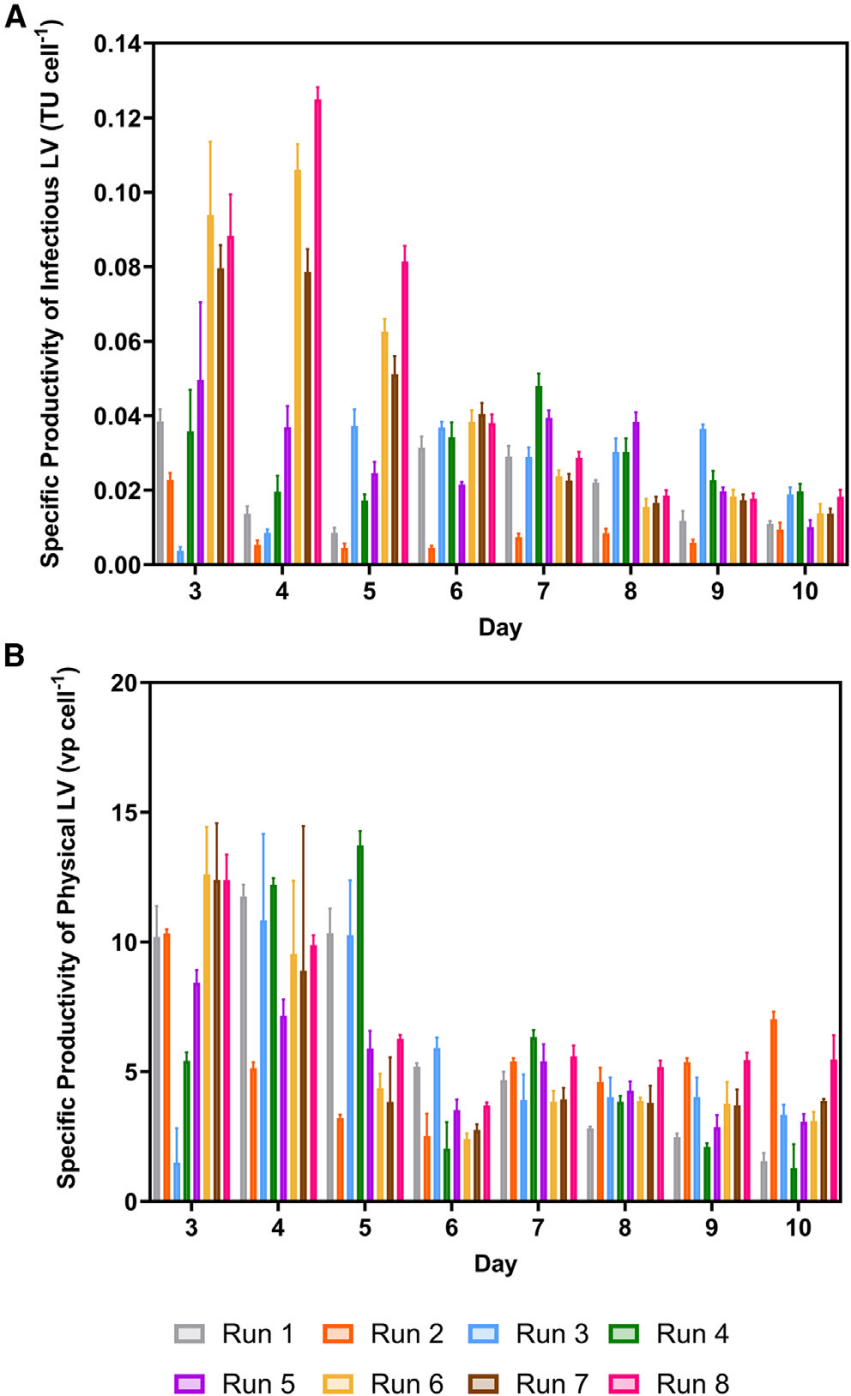
Cell specific lentiviral vector titres Daily specific productivity of (A) infectious and (B) physical LVs during the iCELLis Nano bioreactor runs. Bars represent mean value ± 1 SD.

Since infectious LV titers were maximized at 1.5 VVD, this was selected for further optimization by lowering the culture pH to 6.85 2 days after seeding from 7.20. This increased the infectious LV yield to 9.10 × 10^4^ TU cm^−2^ in run 6. No further increase in physical LV yield was observed. As shown in Figure 3A, the decrease in the pH corresponded with a sharp increase in the specific productivity of infectious LVs on days 3, 4, and 5 of the runs. A similar increase was observed in Figure 3B concerning the physical LV titers at the same time. However, these increases were only maintained until day 5. Beyond this point, the specific productivities of both infectious and physical LVs were similar to the pH 7.20 runs. During the runs at pH 7.20, the specific productivities were similar throughout the process.

Thus, the final process parameters were set at 1.5 VVD and a pH of 6.85. Two additional runs were undertaken using these conditions to assess the batch-to-batch variability (runs 7 and 8). The coefficient of variation (CV) of total infectious and physical LV yields was 6.4% and 10.0%, respectively. The physical-to-infectious particle ratio LVs during these runs was 135 ± 7.8, with a CV of 6.0%.

Concurrent with the bioreactor runs, T-25 control flasks were seeded at 3 × 10^4^ cells cm^−2^ and quasi-perfusion was used to simulate the medium exchange in the bioreactor. Table 4 outlines the LV titers and physical-to-infectious titer ratios achieved during the control experiments. Similar to the bioreactor runs, the 0.5 VVD process in flasks showed the lowest infectious yield of (1.78 ± 0.11) × 10^5^ TU cm^−2^, while medium exchange rates of 1 and 1.5 VVD resulted in the highest infectious LV yields of (4.67 ± 0.09) × 10^5^ TU cm^−2^ and (4.71 ± 0.16) × 10^5^ TU cm^−2^, respectively. The yields of physical LV were consistent across the quasi-perfusion rates. The 0.5 VVD process demonstrated the highest physical-to-infectious titer ratio, consistent with the bioreactor process. In the control flask, the 1 VVD rate produced the highest-quality supernatant, with a ratio of 77 ± 12. With an increase in quasi-perfusion rate from 1 to 2 VVD in the control flasks, the quality of the supernatant decreased, with higher ratios being obtained.

**Table 4:** Infectious and physical LV yields obtained from the control flasks (N = 3)

Quasi-perfusion rate	Total TU	Total vp	Total TU cm^−2^	Total vp cm^−2^	Ratio vp:TU
0.5	(4.44 ± 0.03) × 10^6^	(2.01 ± 0.05) × 10^9^	(1.78 ± 0.11) × 10^5^	(8.06 ± 0.21) × 10^7^	(454 ± 37)
1	(1.17 ± 0.08) × 10^7^	(9.02 ± 0.07) × 10^8^	(4.67 ± 0.09) × 10^5^	(3.61 ± 0.03) × 10^7^	(77 ± 12)
1.5	(1.18 ± 0.04) × 10^7^	(1.24 ± 0.03) × 10^9^	(4.71 ± 0.16) × 10^5^	(4.97 ± 0.01) × 10^7^	(108 ± 42)
2	(5.51 ± 0.02) × 10^6^	(1.36 ± 0.06) × 10^9^	(2.21 ± 0.05) × 10^5^	(5.42 ± 0.25) × 10^7^	(246 ± 21)

The flasks were seeded with 3 × 10^4^ WinPac-RDpro-GFP cells cm^−2^ on day 0, with medium exchange commencing 2 days after seeding for 8 days. For each quasi-perfusion rate, infectious and physical titers were determined with six and three technical repeats, respectively.

### Impact of process development on double-stranded DNA concentration

**Figure 4:**
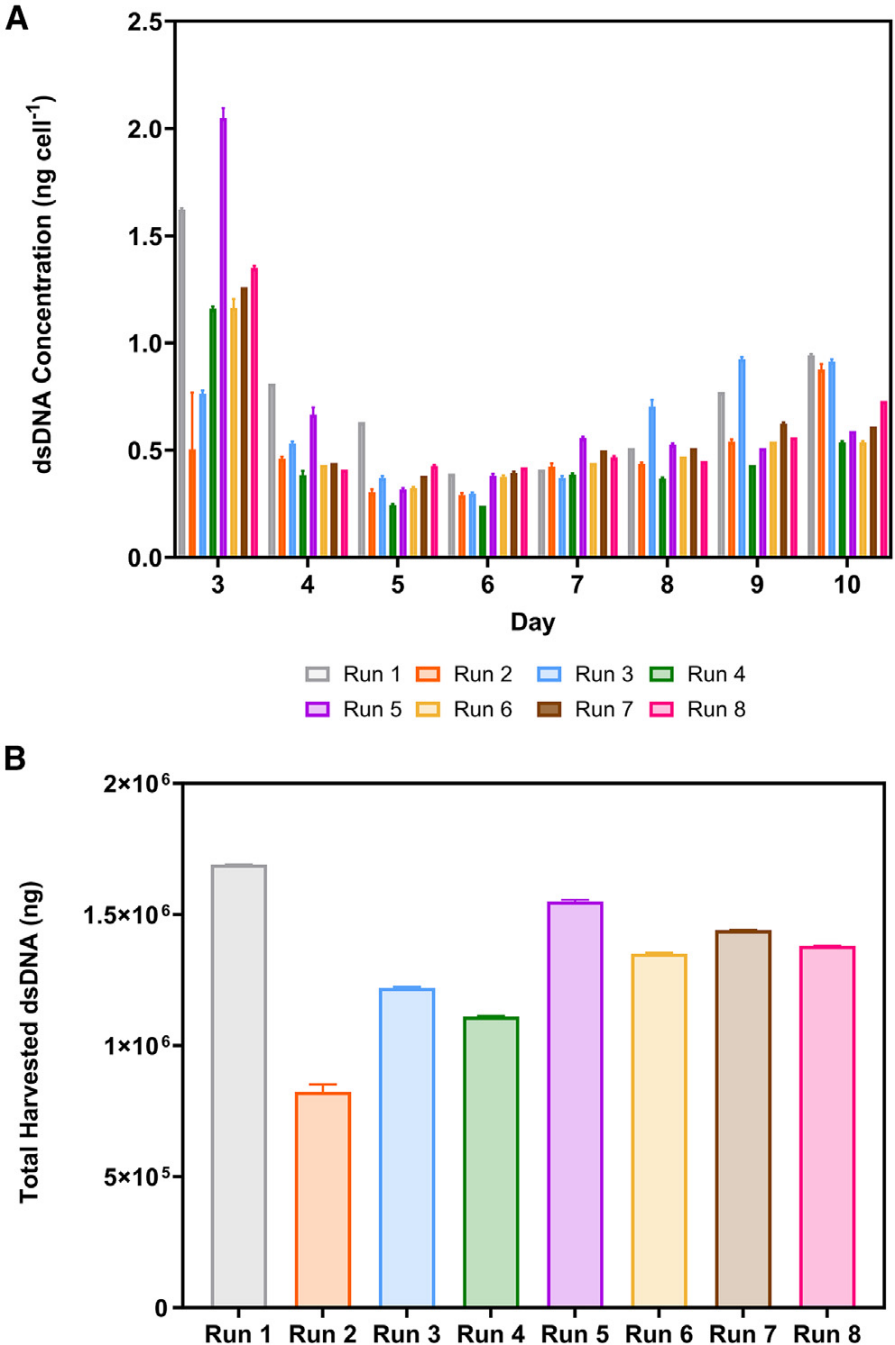
dsDNA concentration Pre-clarification (A) intracellular dsDNA released on a per cell basis and (B) total DNA concentration across the iCELLis Nano bioreactor processes. Bars represent mean value ± 1 SD.

Figure 4A shows the double-stranded DNA (dsDNA) concentration per cell in the perfusate for each day of the bioreactor runs. Across the runs, the highest concentrations of (1.24 ± 0.45) ng cell^−1^ were recorded on day 3, 1 day after perfusion commenced. From day 4 onward, all runs released similar concentrations of intracellular dsDNA at (0.50 ± 0.17) ng cell^−1^. The dsDNA concentration in the culture medium was estimated as (44.62 ± 0.38) ng mL^−1^. Figure 4B displays the total dsDNA in the harvested supernatant. The total harvested dsDNA concentration was the lowest in the 0.5 VVD process at (8.23 ± 0.29) × 10^5^ ng. This is followed by the 1.5 and 1 VVD perfusion processes at pH 7.20 (runs 4 and 3). The highest total dsDNA concentrations were observed in the 1 VVD quasi-perfusion and 2 VVD perfusion processes (runs 1 and 5).

## Discussion

LVs are important for manufacturing gene-modified cell therapies; they facilitate the efficient integration of therapeutic transgenes into the cellular genome, enabling stable transgene expression.^1^ Their manufacture typically involves co-transfection of mammalian cells with plasmid DNA encoding the LV genome.^16^ However, transient transfection poses challenges like batch-to-batch variability and high costs of plasmid DNA and transfection reagents.^17^^,^^18^ Stable producer cell lines enable reproducible, scalable LV manufacturing and decrease process costs by eliminating the need for plasmid DNA and transfection reagents.^17^^,^^19^ This work used the WinPac-RDpro-GFP cell line that constitutively expresses third-generation LVs in a fixed-bed bioreactor to establish a continuous manufacturing process for LVs.^24^ The impact of modulating the perfusion rate and reducing the culture pH was also evaluated.

The iCELLis Nano bioreactor was seeded with 3 × 10^4^ cells cm^−2^ with the linear speed set to 2 cm s^−1^ to promote attachment to PET macro-carriers. Achieving 80% cell attachment took four to 6 h, which was slower than previous reports for adherent human embryonic kidney (HEK) 293T cells in the iCELLis Nano and Scale-X bioreactors.^32^^,^^41^ In these studies, cell attachment of 80% was achieved in less than 1 hour. Possible reasons for this difference include cell modifications to express LVs constitutively. This could have affected integrin expression and resulted in slower adherence to the macro-carriers. Another possible cause is random clonal variation.

The lowest maximum cell densities were observed in the runs with medium exchange rates of 0.5 and 1 VVD. The low medium exchange rates could have impacted cell proliferation by leading to inhibitory metabolite accumulation or nutrient deficiency, resulting in lower cell densities. This was reflected in the longer doubling times ranging from 44.2 to 45.4 h. The shorter doubling times, ranging from 35.2 to 38.9 h, and higher maximum cell densities at perfusion rates of 1.5 and 2 VVD suggested that higher perfusion rates supported more rapid cell expansion. High cell viability (>90%) in all runs indicated that the culture conditions sustained metabolically active cells.

At the end of the processes, it was determined that the cell distribution in the fixed bed was slightly lower at the bottom than at the top. The fixed bed comprises hundreds of PET macro-carriers (13.9 cm^2^ surface area each), forming either a low (96 g L^−1^) or high (144 g L^−1^) compaction.^30^ Previous studies showed that the high compaction (4 m^2^) fixed bed exhibited uneven cell distribution with higher cell densities at the bottom compared with the top.^31^ This variation is attributed to loose carriers and packing, which inherently affect the fixed bed’s consistency. In contrast, the low-compaction fixed bed demonstrated a more uniform cell distribution 72 h after transfection.^31^

Glucose can be catabolized by many metabolic routes, but a considerable proportion is degraded by anaerobic glycolysis, leading to lactate production.^42^ As glucose was not depleted during any process, this was not a cause for the reduced maximum cell densities in runs 1, 2, and 3. The decreased glucose concentration during the low pH runs could indicate that this induced a metabolic shift. The lowest perfusion rate (0.5 VVD) resulted in lactate accumulation exceeding 20 mmol L^−1^ on day 7, but these concentrations are not considered inhibitory to cell expansion. The low lactate concentrations at pH 6.85 runs may indicate reduced production or a shift from production to consumption. This shift has been observed in mammalian cells under reduced culture pH and increased carbon dioxide partial pressure.^43^

The lactate yield from glucose allows the evaluation of cell metabolism. Oxidative phosphorylation is the most efficient glucose consumption method, producing 30–32 ATP molecules per glucose mole.^44^ Aerobic glycolysis yields two ATP molecules and two moles of lactate per glucose mole.^45^ Occasionally, the calculated lactate yield from glucose exceeded the maximum theoretical value of 2 mol mol^−1^, particularly in the first two days. This indicated lactate was produced from alternative carbon sources like glutamine or amino acids.^45^ At a lower culture pH of 6.85, the yield was (0.45 ± 0.15) mol mol^−1^, suggesting a metabolic shift due to the reduced pH or increased carbon dioxide concentrations.

Ammonium accumulates from the chemical degradation of glutamine or the enzymatic action of fetal bovine serum (FBS).^46^^,^^47^ Before medium exchange, there was a sharp increase in ammonium concentration, indicating the degradation of GlutaMAX to produce amino acids. However, the ammonium levels were kept below (1.03 ± 0.01) mmol L^−1^, which is not inhibitory for cell expansion.

The lowest yield of infectious LVs was observed during the 0.5 VVD process (run 2), followed by the quasi-perfusion and perfusion runs at 1 VVD. This is partly attributed to the short half-life of the RDpro-pseudotyped LVs, determined previously as (21.2 ± 8.6) hours.^48^ Another factor is the lower maximum cell densities in these runs compared with higher perfusion rates, possibly due to inhibitory metabolite accumulation or nutrient deficiency. A final possible cause of the low infectious titers was losses due to autotransduction, where the vector transduces the producer cell. The occurrence of autotransduction could be confirmed by determining the copy number per cell for each integrated component using quantitative PCR throughout the bioreactor runs.^49^ This is supported by the 0.5 and 1 VVD runs having the lowest total physical LV yields. Conversely, the increased LV yields observed when raising the perfusion rate to 1.5 VVD can be attributed to improved culture conditions supporting higher cell densities. Additionally, reducing the time the vector spends at 37°C and flowing through the fixed bed and stirrer bar contributed to the increase. The plateau in infectious LV yields at 1.5 VVD is because culture conditions reach the maximum cell density supported by the macro-carriers. Thus, increasing the perfusion rate to 2 VVD or beyond did not yield higher cell densities. However, a further increase in infectious LV could be expected as it lowered the exposure time at 37°C and would have reduced losses to autotransduction. The physical-to-infectious particle ratio reflects the viral preparation quality, with lower ratios indicating a higher proportion of functional vectors.^50^ The low infectious LV yields in the 0.5 VVD process led to the highest ratio, with the higher perfusion rates achieving comparable supernatant qualities. As 1.5 VVD maximized the infectious LV yield, it was selected as the perfusion rate. Another consideration was minimizing the processing volumes to facilitate downstream processing (DSP), with the 1.5 VVD process generating 11.7 L supernatant over the total run compared with 4.5 L, 8.1 L, and 15.3 L at 0.5, 1, and 2 VVD, respectively. The increased process volumes at higher VVDs will burden the initial DSP steps by increasing hold and processing times. This could be minimized by continuously concentrating the perfusate from the bioreactor using single-pass tangential flow filtration.^51^^,^^52^

It has previously been demonstrated that mildly acidic conditions can improve titers for VSV-G and GaLV pseudotyped LVs.^31^^,^^33^^,^^34^^,^^39^ Therefore, whether reducing the culture pH would increase LV yields obtained when using a stable cell line producing RDpro pseudotyped LVs was investigated. Reducing the culture pH to 6.85 from 7.20 at 2 days after seeding resulted in a 1.2-fold increase in infectious LV yield. The increased infectious LV titer could be due to reduced losses through autotransduction. Interestingly, the increased physical and infectious LV production was only observed for approximately 72 h. This could be attributed to the depletion of nutrients or supplements required for higher LV production, increased stress on the cells induced by LV production, or the pH shift only inducing a temporary increase in LV titers. The pH reduction further enhanced the physical-to-infectious titers ratio to 135, consistent with previously reported values.^31^^,^^50^ As 1.5 VVD and pH 6.85 maximized the infectious yield, these were selected as the final parameters.

It was postulated that using a stable producer cell line for LV production would reduce batch-to-batch variability compared with transient transfection. To investigate this, two additional runs at 1.5 VVD and a culture pH of 6.85 were performed. The low batch-to-batch variability observed across the three runs indicated the robustness of the process using the stable producer cell line. Recent work demonstrated the high batch-to-batch variability of different pseudotyped LVs, which were transiently produced in flasks, including RDpro pseudotyped LVs (same envelope protein as in this work).^26^ Furthermore, in comparison with the CVs achieved in this work (6.4% and 10.0% for total infectious and physical titers, respectively), a study using transient transfection of VSV-G LVs in the iCELLis Nano bioreactor resulted in CVs of total infectious and physical titers of 7.9% and 21.2%, respectively.^39^ Although using different pseudotypes, previous work demonstrated that VSV-G LVs have lower batch-to-batch variability than RDpro LVs.^26^ The improvement in batch variability in the bioreactor work for RDpro, compared with VSV-G, is attributed to using a stable producer cell line.

The lowest infectious LV yields were observed at 0.5 VVD in the control flasks. As with the bioreactor, this is likely due to the short vector half-life at 37°C and losses through autotransduction. Conversely, the increased infectious LV observed at high quasi-perfusion rates of 1 and 1.5 VVD can be attributed to the shorter vector exposure times in the flask. The plateau at 2 VVD could be caused by the higher culture pH in those vessels, associated with lower infectious LV titers.

dsDNA is a significant process-related impurity that requires removal during DSP. In the stable cell line process, dsDNA originates from producer cell lysis and FBS supplementation of the medium. The dsDNA concentration per-cell in the perfusate was highest a day after perfusion began. This can be attributed to the cells being within the bioreactor for 48 h before perfusion commenced and from the death of cells that failed to attach during the inoculation phase, where the linear speed through the vessel was higher at 2 cm s^−1^. The similar released dsDNA concentrations observed from days 4 to 10 are supported by the high cell viability observed throughout the processes. The lowest total dsDNA concentration was observed during the 0.5 VVD process. The FBS-supplemented culture medium also contains dsDNA, so lower medium exchange rates will result in lower total concentrations. The higher concentrations observed at the increased medium exchange rates can be attributed to adding additional dsDNA by increased medium consumption. Additionally, as these runs also produced higher LV titers, this could have induced cell death by genotoxicity due to exposure of the producer cells to high vector MOIs over the extended culture.^49^ This would have also increased the intracellular dsDNA release.

In conclusion, this work established a continuous process for manufacturing LVs using a stable producer cell line in a fixed-bed bioreactor. This approach provided extended production periods and eliminated the need for costly plasmid DNA or transfection reagent. Increasing the perfusion rate resulted in higher infectious LV yields, attributed to improved culture conditions that promoted cell growth and reduced vector exposure in the bioreactor. Mildly acidic conditions also resulted in increased infectious LV titers. The process development led to a more than 2-fold increase in LV yields, potentially more than double the number of doses supplied, compared with the 1 VVD quasi-perfusion process. The continuous process generated large volumes of high-quality vector-containing supernatant based on the physical-to-infectious LV supernatant. The process could be scaled approximately 8-fold in the same platform and even further in the iCELLis 500 bioreactor. Using a stable cell line simplified manufacturing by eliminating the need for optimizing various parameters required in transfection-based methods, such as transfection reagent, plasmid ratios, the DNA-to-transfection reagent ratio, and cell density at transfection. The process demonstrated low-batch-to-batch variability, which facilitates DSP.

Future studies could aim to increase infectious LV titers by exploring culture medium supplementation, assessing the effects of scaling the process on performance, or transitioning from a fixed perfusion rate to one modulated based on online measurement of a particular process parameter. Additionally, the bioreactor could be integrated with continuous DSP unit operations, such as single-pass tangential flow filtration or chromatography.

## Materials and methods

### Cell culture

WinPac-RDpro-GFP cells were cultured in DMEM modified with high glucose, GlutaMAX and phenol red (Thermo Fisher Scientific) and supplemented with 10% volume/volume (v/v) FBS (Gibco [Thermo Fisher Scientific]) in a humidified incubator at 37°C and 5% CO_2_. During cell expansion, blasticidin, hygromycin, phleomycin, and puromycin (InvivoGen, Inc.) were present at 10, 100, 30, and 1 μg mL^−1^ working concentrations, respectively. However, these antibiotics were removed during LV production.

HEK 293T cells (ATCC) were cultured at 37°C and 5% CO_2_ in DMEM modified with high glucose, GlutaMAX, and phenol red supplemented with 10% (v/v) FBS.

### LV production in iCELLis Nano bioreactor

The iCELLis Nano bioreactor, equipped with a 2 cm low-compaction fixed-bed (0.53 m^2^ surface area) and the mPath bioreactor benchtop control tower (Cytiva), was used. The vessel, filled with 600 mL of growth medium, underwent an overnight equilibration at 37°C with pH and dissolved oxygen (DO) setpoints of 7.20 ± 0.05 and 50% ± 2%, respectively. Inoculation occurred the following day at a seeding density of 3 × 10^4^ cells cm^−2^, with the total vessel volume increasing to 0.17 mL cm^−2^ (900-mL working volume). Linear speed was initially set at 2 cm s^−1^ for cell attachment, monitored hourly, and reduced to 1 cm s^−1^ once 80% cell attachment was achieved. DO was tracked with a VisiFerm DO ECS 120 H0 (Hamilton Company, Inc.), and pH was regulated using carbon dioxide and 7.5% (v/v) sodium bicarbonate (Merck KGaA). Daily offline pH measurements were used for calibration. Macro-carriers were sampled daily from the fixed bed, placed in 2 mL Eppendorf Tubes with 1.5 mL lysis solution A100 (ChemoMetec A/S), and vortexed before counting lysed nuclei. Medium exchange began at 2 days after seeding through perfusion culture or manual exchange. For low pH runs, the pH was adjusted to 6.85 ± 0.05 at 2 days after seeding. The perfused medium was collected, pooled, and stored at room temperature. Once per day, approximately 20 mL was removed from the pooled perfusate to determine the infectious titer and to aliquot and stored at −80°C to determine metabolite concentration, physical titer, and dsDNA concentration.

Simultaneously, T-25 flasks (Thermo Fisher Scientific) were seeded with 3 × 10^4^ WinPac-RDpro-GFP cells cm^−2^ and 0.17 mL cm^−2^ of culture medium. Quasi-perfusion started 2 days after seeding at rates of 0.5, 1, 1.5, and 2 VVD to mimic the bioreactor perfusion rate. Samples were retained at −80°C for physical LV titration.

### LV quantification

Infectious LV titers were assessed by flow cytometric detection of GFP-positive cells. A sample of the pooled LV perfusate was taken each day and assayed with six technical replicates. HEK293T cells were seeded at 3 × 10^5^ cells per well in 12-well plates (Thermo Fisher Scientific) and transduced with LV neat samples in the presence of 8 μg mL^−1^ polybrene (Santa Cruz Biotechnology, Inc.) in a total volume of 500 μL. After 24 h of incubation, 1 mL medium was added to each well, followed by trypsinization and staining with 7-AAD (eBiosciences, Inc.) at 72 h after transduction. Flow cytometric analysis used a BD LSRFortessa Cell Analyzer (Becton, Dickinson and Company). Infectious titers were computed using Equation 1 based on vector dilutions, where 1%–20% of the live cell population was GFP positive.

(Equation 1) $$Infectious\;titre\;\left(TU.{mL}^{-1}\right)=\left\{Number\;of\;cells\;at\;transduction\times \frac{\left(\%Live\;GFP-positive\;cells/100\right)}{Vector\;input\;volume}\right\}\times Dilution\;factor\text{.}$$

The physical vector particles per milliliter (vp mL^−1^) were quantified by measuring HIV-1 p24 capsid protein through an enzyme-linked immunosorbent assay (OriGene Technologies, Inc.) using the recommended conversion of 10,000 physical LV particles per 1 pg of p24. Each sample underwent assessment with three technical repeats.

### Cell count and viability measurement

Cell concentration and viability were assessed through a NucleoCounter NC-200 system and Via1-Cassette (ChemoMetec A/S) following the “Viability and Cell Count Assay” protocol. If needed, cells were diluted with growth medium to achieve the recommended concentration range of 5 × 10^4^ to 5 × 10^6^ cells mL^−1^.

### Metabolite analysis

Samples were preserved in triplicate and kept at −80°C. Ammonium, glucose, and lactate concentrations were analyzed using the CuBiAn Bioanalyzer (Optocell GmbH & Co. KG). The operation of the system followed the guidelines provided by the manufacturer.

### Offline pH measurement

Offline pH was measured using a Seven-Compact pH meter S220 (Mettler Toledo, LLC).

### Determination of dsDNA concentration

dsDNA was quantified using the Quant-iT PicoGreen dsDNA assay kit (Thermo Fisher Scientific). The ***λ*** dsDNA standard was diluted with 1× TE buffer across 0–1,000 ng mL^−1^ concentrations. In 96-well plates, 100 μL Quant-iT PicoGreen dsDNA reagent was mixed with 100 μL of the sample or standard, followed by a 5-min incubation at room temperature. Fluorescence was measured at 480/520 nm using a CLARIOstar plate reader (BMG LABTECH GmbH). DNA concentrations were determined using the generated standard curve, with all measurements performed in triplicate.

### Graphing and statistical analysis

GraphPad Prism v9 (GraphPad Software, LLC) was used for graphical representation. In-text values are reported as the mean ± 1 SD.

### Equations

#### Specific growth rate

The specific growth rate (μ) was computed using Equation 2, with Cx(t) and Cx(0) denoting the total cell numbers at the end and the start of the exponential growth phase, respectively, and time represented in hours:

(Equation 2) $$\mu =\frac{\ln\left(\frac{Cx\left(t\right)}{Cx\left(0\right)}\right)}{\Delta t}\text{.}$$

#### Doubling time

The doubling time (t_d_) was determined using Equation 3, with μ denoting the specific growth rate (h^−1^):

(Equation 3) $$t_{d}=\frac{\ln\left(2\right)}{\mu }\text{.}$$

#### Population doublings

Population doublings were computed using Equation 4, with Cx(t) and Cx(0) indicating the total cell numbers at the end and the start of the exponential growth phase, respectively:

(Equation 4) $$P_{d}=\frac{1}{\ln\left(2\right)}\times \ln\left(\frac{C_{x}\left(t\right)}{C_{x}\left(0\right)}\right)\text{.}$$

#### Lactate yield from glucose

Lactate yield from glucose (YLac|Glc) was determined using Equation 5, with Δ[Lac] and Δ[Glc] indicating the variations in lactate and glucose concentrations over the same time:

(Equation 5) $$\frac{Y_{Lac}}{Y_{Glc}}=\frac{\Delta \left[Lac\right]}{\Delta \left[Glc\right]}\text{.}$$

#### CV

The CV was computed using Equation 6, with σ representing the population’s SD, and μ being the population mean:

(Equation 6) $$CV=\frac{\sigma }{\mu }\times 100\text{.}$$

## Data and code availability

All data generated or analyzed during this study are included in this published article.

## Supplemental information

Document S1. Figure S1

Document S2. Article plus supplemental information
