# Supplementary material for: Continuous manufacturing of lentiviral vectors using a stable producer cell line in a fixed-bed bioreactor
Source: Mol Ther Methods Clin Dev. 2024 Feb 9;32(1):101209. doi: 10.1016/j.omtm.2024.101209 (PMC10907162; doi:10.1016/j.omtm.2024.101209)
Supplement: Document S1. Figure S1 [file mmc1.pdf]

OMTM, Volume *32*

Supplemental information

Continuous manufacturing of lentiviral vectors using a stable producer cell line in a fixed-bed bioreactor

Dale J. Stibbs, Pedro Silva Couto, Yasuhiro Takeuchi, Qasim A. Rafiq, Nigel B. Jackson, and Andrea C.M.E. Rayat

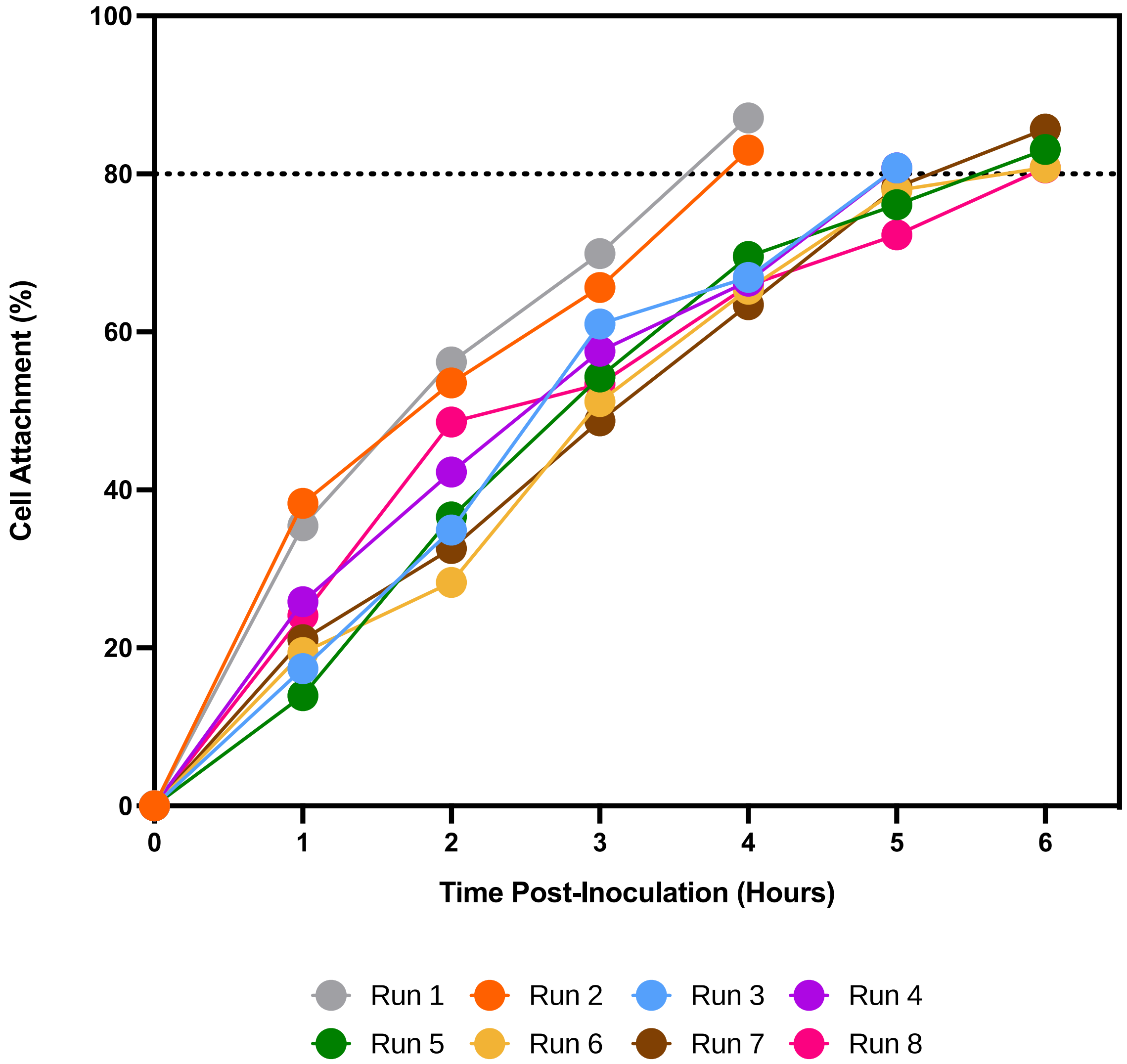

Figure S1 – Progression of cell attachment in the iCELLis Nano bioreactor after inoculation with 3 x 10^4 cells.cm^{-2}. The linear speed was initially set at 2 cm s^{-1} to promote cell attachment. The line at 80% attachment represents the point at which the linear speed was reduced to 1 cm s^{-1}.
